# Practice variation in surgical treatment for lumbar degenerative disc disease: exploring regional and hospital factors influencing surgical rates

**DOI:** 10.1038/s41598-024-59629-9

**Published:** 2024-04-23

**Authors:** Juliëtte J. C. M. van Munster, Ilan J. Y. Halperin, Frank H. Ardesch, Wilbert B. van den Hout, Peter Paul G. van Benthem, Wouter Moojen, Wilco C. Peul

**Affiliations:** 1 University Neurosurgical Centre Holland (UNCH), LUMC | HMC | HAGA, Leiden & The Hague, the Netherlands; 2 Department of Otorhinolaryngology and Head and Neck Surgery, Leiden University Medical Center, Leiden University, Leiden, the Netherlands; 3 Department of Public Health and Primary Care, Health Campus The Hague, Leiden University Medical Center, The Hague, The Netherlands; 4 Department of Biomedical Data Sciences, Leiden University Medical Center, Leiden, the Netherlands; 5 Department of Neurosurgery, Leiden University Medical Centre, P.O. Box 9600, 2300 RC Leiden, the Netherlands

**Keywords:** Neuromuscular disease, Health policy, Health services

## Abstract

The presence of significant, unwarranted variation in treatment suggests that clinical decision making also depends on where patients live instead of what they need and prefer. Historically, high practice variation in surgical treatment for lumbar degenerative disc disease (LDDD) has been documented. This study aimed to investigate current regional variation in surgical treatment for sciatica resulting from LDDD. We conducted a retrospective, cross-sectional analysis of all Dutch adults (>18 years) between 2016 and 2019. Demographic data from Statistics Netherlands were merged with a nationwide claims database, covering over 99% of the population. Inclusion criteria comprised LDDD diagnosis codes and relevant surgical codes. Practice variation was assessed at the level of postal code areas and hospital service areas (HSAs). Multivariable logistic regression analysis was employed to identify variables associated with surgical treatment. Among the 119,148 hospital visitors with LDDD, 14,840 underwent surgical treatment. Practice variation for laminectomies and discectomies showed less than two-fold variation in both postal code and HSAs. However, instrumented fusion surgery demonstrated a five-fold variation in postal code areas and three-fold variation in HSAs. Predictors of receiving surgical treatment included opioid prescription and patient referral status. Gender differences were observed, with males more likely to undergo laminectomy or discectomy, and females more likely to receive instrumented fusion surgery. Our study revealed low variation rates for discectomies and laminectomies, while indicating a high variation rate for instrumented fusion surgery in LDDD patients. High-quality research is needed on the extent of guideline implementation and its influence on practice variation.

## Introduction

Clinical decision making is expected to be grounded in high-quality scientific evidence. However, the presence of significant, unwarranted variation in treatment suggests that decision making is also influenced by patients' geographical location rather than their individual needs and preferences^1^. Hence, practice variation might partly be driven by differences in physicians’ beliefs about the effectiveness of the treatment. Previous studies have reported substantial regional variation in surgical rates for Lumbar Radicular Syndrome (LRS) caused by degenerative disc disease (DDD) within and between regions and countries^2^.

LRS, resulting from degenerative disc disease, can be attributed to a herniated disc, spinal stenosis, or spondylolisthesis. It is a prevalent disorder in routine healthcare practice leading to disability, sick leave, and high societal and healthcare cost^3,4^. High-quality evidence on the effectiveness and timing of surgical treatment for  low back disorders improved significantly in the last decades^5–8^. In the Netherlands, recommendations from high-quality research have been incorporated into new evidence-based guidelines published first in 2008^9,10,13^. These guideline recommendations have further been integrated into the "Wise choices concerning a lumbar disc herniation" initiative as part of the Dutch "Choosing Wisely Campaign." However, the quality of evidence regarding the effectiveness of instrumented fusion surgery in patients with degenerative spondylolisthesis, degenerative low back pain or recurrent herniation is notably limited^9^. This was also stated in the 2019 Knowledge Agenda of the Dutch Association of Neurosurgeons (NvVN).

Previously, we observed a decline in the number of procedures for lumbar disc herniations in the Netherlands between 2007 and 2020^11^. Consequently, we are now interested in investigating the current regional variation in surgical treatment for sciatica resulting from lumbar degenerative disc disease. Given the availability of evidence-based guidelines, we anticipate observing low variation^12^. Furthermore, we hypothesize that there will be lower variation in laminectomies and discectomies for lumbar DDD compared to instrumented fusion surgery due to the presence of high-quality evidence supporting the former procedures and the lack of evidence supporting the latter^9,10,13^. Secondary, we aim to explore the influence of both hospital and patient factors in explaining the observed regional variation in surgical rates.

## Methods

### Data sources and population

For the purpose of this study, we utilized and merged two databases. Firstly, we accessed a nationwide claims database provided by VEKTIS. This database collects claims data from all healthcare insurance companies in the Netherlands, ensuring comprehensive coverage of over 99% of the Dutch population. It has been determined to be over 95% accurate when compared to hospital records^14^. Secondly, we obtained a database from Statistics Netherlands (CBS), which includes detailed patient characteristics^15^. The VEKTIS database contained data on all Dutch individuals aged 18 years and older who sought hospital care for lumbar degenerative disc disease between January 1, 2016, and December 31, 2019. The CBS databases encompassed information on all Dutch inhabitants aged 18 years and older. CBS data was provided through the Extramural LUMC Academic Network (ELAN) data infrastructure project^16^.

### Definitions and variables

In the VEKTIS database, we extracted specific data related to lumbar degenerative disc disease. This included diagnosis codes that are associated with sciatica, as well as surgical care products for discectomy, laminectomy, and fusion surgery (Supplemental files, Tables S1, S2). To ensure consistency in the registration process, we used multiple codes to capture indications for surgical treatment, minimizing potential variation in registration among physicians. We excluded patients who had cervical degenerative disc disease, spinal infections, fractures or trauma, malignancies, or congenital diseases. Additionally, individuals who had undergone back surgery within the past year were excluded from the analysis.

To account for physician registration differences and their potential impact on surgical rates, we also combined data on laminectomies and discectomies. We categorized patients into two age groups: those younger than 55 years and those older than 54 years. This distinction allowed us to differentiate between lumbar disc herniations, which are more prevalent in younger individuals, and lumbar spinal stenosis, which is commonly observed among the elderly. We separately analyzed the rates of instrumented fusion surgery.

We collected additional information on opioid prescriptions, which were recorded during the same year as the Diagnosis Treatment Combination (DBC) code registration. These prescription data were based on mandatory basic health insurance records for all Dutch inhabitants. Furthermore, we obtained details on lumbar injection therapy, including the corresponding DBC codes and procedure codes (found in the supplemental files). Lastly, we gathered data on the type of hospital (general hospital, teaching hospital, university hospital, private clinic), and the number of hospitals visited (indicating referrals from other hospitals if more than one).

From the CBS database, we extracted patient-specific information, including age, sex, comorbidities assessed using the Charlson Comorbidity Index, ZIP code, average household income, level of education (categorized as low, middle, high), type of income (employer, employee, retired, student, unemployed), and migration background (whether they were born in the Netherlands or another country).

### Calculation of indirect adjusted surgical rates

To account for potential confounding factors, surgical rates were adjusted for age, sex, Charlson Comorbidity Index (CCI), household income, and level of education^17^. Advanced age is associated with increased incidence of spine disease, while females and individuals with lower socioeconomic status exhibit an elevated likelihood of experiencing suboptimal outcomes following sciatica^18–20^. Moreover, patients with comorbidities face an elevated risk of perioperative complications, prompting a hesitancy among surgeons towards surgical interventions^21^. The above mentioned factors are also associated with regional variation in the use of surgery in general^12^. Adjusted rates were calculated per 10,000 inhabitants in two-digit postal code areas and per 10,000 patients in neurosurgical spine clusters, which represent hospital service areas (HSAs) in the Netherlands. Referrals within these clusters were considered in the analysis. Private clinics were analyzed separately. The calculation of indirectly adjusted surgical rates per 10,000 inhabitants or hospital visitors followed the following formula:

$$\begin{aligned} &\frac{{Observed \;number \;of \;procedures \;in \;HSA, \;Postal \;code \;area \;or \;neurosurgical \;cluster }_{z}}{{Expected \;number \;of \;procedures \;in \;HSA, \;Postal \;code \;area \;or \;neurosurgical \;cluster}_{z}}\\ &\times mean \;procedure \;rate \;per \;\mathrm{10,000} \;inhabitants \;or \;per \;1000 \,hospital \,visitors \end{aligned}$$

Hereafter, we will refer to surgical or referral rates per 10,000 inhabitants in two-digit postal code areas as ‘regional surgical rates’ and to surgical rates per 10,000 inhabitants in neurosurgical clusters as HSA surgical rates.

### Analysis

For all rates, we calculated the Extreme Quotient (EQ, Highest/Lowest rate), EQ_5-95_ (95th percentile/5th percentile), the interquartile range (IQR, 75th percentile/25th percentile), the Coefficient of Variation (CoV, Mean/SD (Standard Deviation)), the Systematic Component of Variation (SCV). The SCV estimated the systematic variation between areas that cannot be account for by the random variation within each area and is calculated by the following formula:

$$\mathrm{SCV }=\frac{1}{N} \left({\sum }_{k=1}^{i}\frac{{(Oi-Ei)}^{2}}{{Ei}^{2}} - {\sum }_{k=1}^{i}\frac{1}{Ei}\right) \times 100$$

An SCV below 5 was considered ‘low variation’, an SCV between 5 and 10 was considered high variation, and an SCV of 10 or higher was considered very high variation^22^.

We utilized mixed-effects logistic regression analyses to investigate the factors underlying the observed variation in regional surgical rates and HSA surgical rates. The analysis of regional rates included patient characteristics such as age, sex, CCI, level of education, household income, type of income, and migration background. For the HSA rates analysis, we further considered variables including referral from another hospital (referred patients), referral center (which were indicated by authors and neurosurgeons WP and WM), neurosurgical regions, prescription of opioids, and lumbar injection therapy. Two-digit postal code areas and HSAs were treated as random effects in the analyses. We opted against the inclusion or examination of interaction terms for variables, primarily due to the extensive size of our database and challenges associated with interpreting outcomes when incorporating such terms. It is important to note that we acknowledge the potential influence of this decision on the odds ratio.

### Analytic approach

All analyses were conducted at the patient level, ensuring that each patient was included only once per year. Continuous variables were presented as mean (SD) or median (IQR) for nonparametric data. Missing data were examined, and multiple imputation with 10 imputation sets was employed to address missing values. Age, sex, CCI, level of education, household income, and postal code area were utilized as predictors. Statistical significance was defined as p-value < 0.05. SPSS Statistics (version 26) was employed for all statistical analyses, while Python was used to generate maps illustrating the two-digit postal code areas.

### Ethical approach

The current study adheres to the principles outlined in the Declaration of Helsinki. Our study protocol (N20.075) underwent thorough review and approval by the Medical Ethics Committee Leiden Den Haag and Delft. The committee determined that official approval was not required because participants were not directly involved in the study and patient anonymity was safeguarded in the database. Thereby, the informed consent was waived by the Medical Ethics Committee of Leiden Den Haag and Delft.

## Results

### Study population

The Netherlands had an average population of 13.8 million adults aged 18 years or older between 2016 and 2019 (Table 1). Among them, we identified 119,148 adult individuals who visited the hospital annually for lumbar degenerative disc disease (Table 2). The annual number of procedures ranged from 14,348 to 15,492. Detailed information on specific diagnoses and procedures can be found in Table 1 of the supplemental files. The overall rate of missing data for all variables was less than 5%, except for the variable 'level of education,' which had 41% missing data. The missing data were determined to be missing at random and were imputed as described in the methods section.

**Table 1:** Baseline characteristics for our study population between 2016 and 2019.

Variable	All inhabitants (n = 13,795,549)	Lumbar DDD patients (n = 119,148)	Surgical patients (n = 14,840)
Age (years), mean (SD)	49.5 (18.8)	57.5 (15.6)	58.1 (15.4)
Sex, n (%)			
Male	6,798,912 (49)	54,686 (46)	7565 (51)
Female	6,996,637 (51)	64,462 (54)	7275 (49)
Comorbidities, n (%)			
0	116,99,719 (85)	83,156 (70)	10,386 (70)
1	838,777 (6)	14,339 (12)	1725 (12)
2	787,544 (6)	11,725 (10)	1453 (10)
≥ 3	469,509 (3)	8936 (8)	1286 (9)
Level of education, n (%)			
Low	3,587,100 (26)	40,986 (34)	5007 (34)
Middle	5,295,067 (38)	44,289 (37)	5633 (38)
High	4,913,382 (36)	33,873 (28)	4200 (28)
Household income, median (IQR)	27,182 (19,406–36,279)	25,938 (19,197–34,552)	27,786 (25,893–36,471)
Occupation, n (%)			
Employee	6,423,232 (47)	43,710 (37)	5700 (38)
Employer or entrepreneur	1,209,288 (9)	8280 (7)	920 (6)
Retired	3,183,242 (23)	42,160 (35)	5553 (37)
Unemployed	1,866,463 (14)	23,639 (20)	2518 (17)
Student	1,113,324 (8)	1359 (1)	149 (1)
Migration background, n (%)			
Yes	2,103,826 (15)	17,566 (15)	1427 (10)
No	11,691,723 (85)	101,582 (85)	13,431 (90)

**Table 2:** Baseline characteristics of hospital visitors for lumbar DDD between 2016 and 2019.

Variables	Lumbar DDD^a^ patients (n = 119148^b^)	Surgical patients (n = 14840^b^)
Hospital type, n (%)		
University hospital	3962 (3)	609 (4)
Teaching hospital	55,603 (47)	7338 (49)
General hospital	43,744 (37)	4331 (29)
Private clinic	15,839 (13)	2563 (17)
Lumbar injection, n (%)		
Yes	17,721 (15)	2338 (16)
No	101,427 (85)	12,502 (84)
Prescription of opioids, n (%)		
Yes	54,850 (46)	9985 (67)
No	64,298 (54)	4856 (33)
Referred patients^c^, n (%)	10,116 (8)	4261 (29)
Referral center, n (%)	17,987 (15)	3586 (24)

^a^Degenerative disc disease.

^b^Average per year.

^c^Referral between hospitals.

### Variation in surgical rates

**Figure 1:**
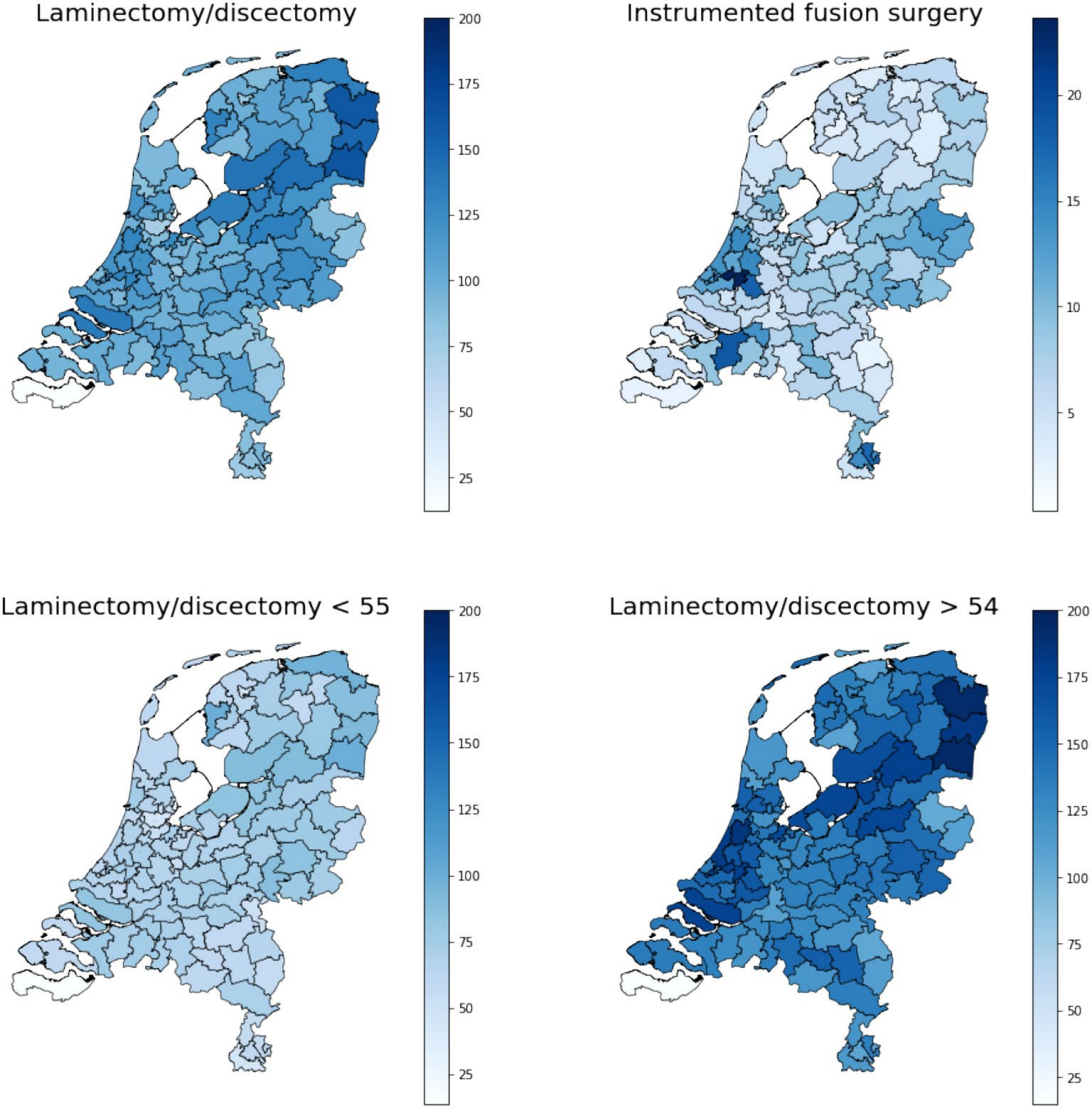
Surgical rates per 100,000 inhabitants in 2-digit postal code areas in the Netherlands.

**Figure 2:**
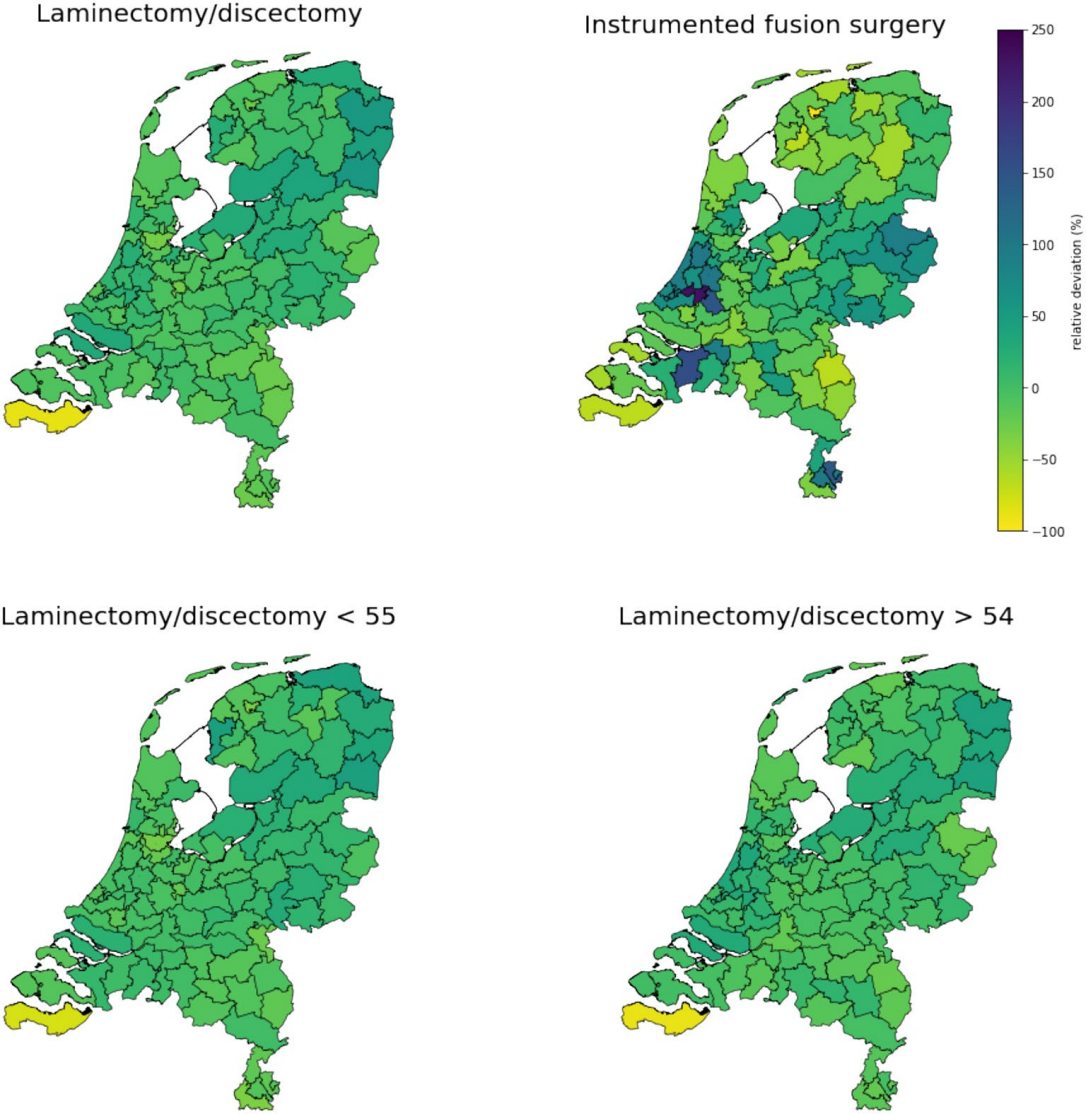
Relative deviation (in percentages) of surgical rates compared to the median rate per 100,000 inhabitants in 2-digit postal code areas in the Netherlands.

**Figure 3:**
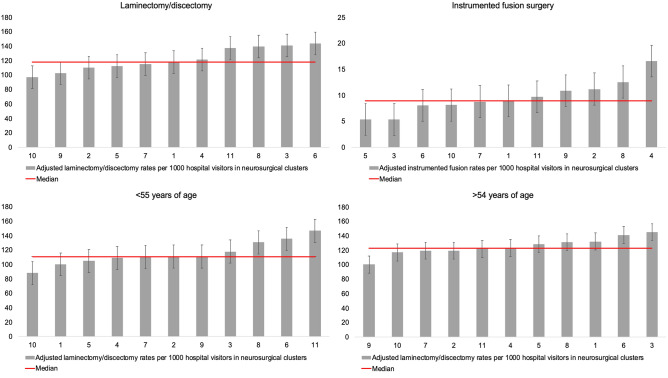
Surgical rates per 1000 hospital visitors in neurosurgical clusters. Bars represents the 11 HSAs in the Netherlands. The red line represents the median surgical rate.

On average, 108 per 100,000 inhabitants age 18 years or older received surgery for lumbar DDD annually between 2016 and 2019. Higher surgical rates were observed in patients aged 55 years and older compared to the younger age group (Fig. 1). Furthermore, laminectomy and discectomy rates were much higher than instrumented fusion surgical rates. Figure 1 and Fig. 2 show that postal code area ‘45’ (which is the white area in the southwest on the geographic maps) has a very low surgical rate, which has a huge impact on the extreme quotient for regional differences. For laminectomy/discectomy rates, low variation was observed between 2016 and 2019 in postal code areas as well as neurosurgical clusters, whereas very high variation was observed for instrumented fusion rates in postal code areas and high variation in neurosurgical clusters as shown by the SCV (Table 3, Fig. 3). Removing outliers, as shown by the EQ_5-95_ in Table 3, surgical rates in both postal code areas and neurosurgical clusters differ more than twofold for instrumented fusion surgery. Surgical rates for laminectomy and discectomy differed between 1.3- and 1.8-fold when removing outliers.

**Table 3:** Practice variation outcomes on surgical rates between 2016 and 2019.

	Average rate^a^	EQ^b^	EQ_5-95_	IqR^c^	CoV^d^	SCV^e^
Laminectomy/discectomy						
Regional surgical rates^f^	105	13.5	1.8	1.2	0.2	3.9
Regional surgical rates						
Patients age < 55 years	69	7.3	1.7	1.2	0.2	2.8
Regional surgical rates						
Patients age > 54 years	139	13.1	1.6	1.2	0.2	2.7
HSA surgical rates^g^	122	1.5	1.4	1.2	0.1	1.5
HSA surgical rates						
Patients age < 55 years	115	1.7	1.5	1.2	0.1	1.6
HSA surgical rates						
Patients age > 54 years	126	1.4	1.3	1.1	0.1	0.7
Instrumented fusion surgery						
Regional surgical rates	8	69	4.6	1.9	0.5	19.9†
HSA surgical rates	10	3.3	2.7	1.3	0.3	8.6*

*High variation.

^†^Very high variation.

(SCV > 3 = likely be due to differences in practice style or ‘medical discretion’; 5–10 = high variation; > 10 = very high variation).

^a^For regional surgical rates: average rate per 100,000 inhabitants. For HSAs: average rate per 1000 hospital visitors.

^b^Extreme Quotient.

^c^Interquartile Range.

^d^Coefficient of Variation.

^e^Systematic Component of Variation.

^f^Per 10,000 inhabitants.

^g^Per 10,000 hospital visitors.

### Explanatory analyses

The risk of receiving a laminectomy or discectomy was 30% lower for female patients compared to male patients (Table 4). Furthermore, the risk was 30% lower for welfare recipients compared to employees, 20% lower for employers, and 10% lower for students. Patients with a migration background had a 40% lower risk of receiving laminectomy or discectomy. Furthermore, the odds of receiving a laminectomy or discectomy were 2.5 times higher for patients who received an opioid prescription and 5.5 times higher for referred patients. The risk of receiving laminectomy or discectomy was 30% lower for patients who already had a lumbar epidural injection.

**Table 4:** Odds ratios for patient and hospital factors associated with surgical treatment.

Variable	Laminectomy and discectomy		Instrumented fusion surgery	
	Odds Ratio (95% CI)	p-value	Odds Ratio (95%CI)	p-value
Patient factors				
Age	1.0 (1.0–1.0)	0.35	1.0 (1.0–1.0)	< 0.001*
Female (compared to male)	0.7 (0.7–0.8)	< 0.001*	1.2 (1.1–1.2)	0.05*
CCI^a^	1.0 (1.0–1.0)	0.01*	1.0 (1.0–1.0)	0.04*
Household income	1.0 (1.0–1.0)	0.32	1.0 (1.0–1.0)	0.34
Employer^b^	0.8 (0.7–0.8)	< 0.001*	0.8 (0.7–1.0)	0.17
Student^b^	0.9 (0.7–1.1)	0.56	1.0 (0.6–1.8)	0.80
Retired^b^	1.1 (1.0–1.2)	0.10	0.7 (0.6–0.8)	0.01*
Welfare recipients^b^	0.7 (0.7–0.8)	< 0.001*	1.0 (0.9–1.1)	0.48
Highest education level^c^	1.0 (1.0–1.1)	0.49	1.0 (0.8–1.1)	0.31
Intermediate education level^c^	1.0 (1.0–1.1)	0.39	1.0 (0.9–1.1)	0.29
Migration background	0.6 (0.6–0.7)	< 0.001*	0.6 (0.6–0.7)	< 0.001*
Hospital factors				
Opioid prescription	2.5 (2.5–2.5)	< 0.001*	4.5 (4.0–5.0)	< 0.001*
Referred patients	5.5 (5.5–6.1)	< 0.001*	3.0 (2.7–3.3)	< 0.001*
Referral center	4.5 (0.9–22.2)	0.08	13.5 (1.8–99.5)	0.02*
Lumbar epidural injection	0.7 (0.7–0.8)	< 0.001*	0.6 (0.6–0.7)	< 0.001*

*Statistically significant.

^a^Charlson Comorbidity Index.

^b^Compared to employees.

^c^Compared to lowest education level.

The odds of receiving instrumented fusion surgery was 1.2 times higher for females compared to males. Moreover, retired patients had a 30% lower risk of receiving instrumented fusion surgery compared to employees, whereas patients with a migration background had a 40% lower risk. The odds of receiving instrumented fusion surgery were 4.5 times higher for patients who received an opioid prescription, 3.0 times higher for referred patients, and 13.5 times higher for patients in a referral center. Lastly, patients who already received a lumbar epidural injection had a 40% lower risk of receiving instrumented fusion surgery.

## Discussion

This study analyzed practice variation outcomes for surgical treatment of lumbar DDD as a part of a quality-of-care cycle. Low variation in adjusted surgical rates between regions was expected due to widely available national evidence-based guidelines and efforts to implement the guidelines.

### Summary of principal outcomes

This study showed overall low variation for discectomies and laminectomies in patients with lumbar DDD in 2-digit postal code areas as well as in HSAs. We found low variation in both the younger age group (more likely suffering from HNP) and the older age group (more likely suffering from lumbar stenosis). However, high variation was observed for instrumented fusion surgical rates in 2-digit postal code areas and in HSAs. Surgical rates were influenced by both patients and hospital factors, where patient’s referral status seemed to be the most important factor for both discectomies/laminectomies and instrumented fusion surgery. Prescription of opioids was another important predictor for receiving surgery. It is noticeable that the risk of receiving laminectomy and discectomy was significantly higher for males, whereas the risk of receiving instrumented fusion surgery was higher for females.

### Strengths and limitations

The use of administrative data helped us performing a population-based study containing data from all inhabitants in the Netherlands over a timeframe of four years. However, due to the use of administrative data, it was impossible to include disease severity, disease symptoms (i.e. cauda equina syndrome), and patient outcomes. Also, specific indications for surgery remain unclear. We hope that analyzing age groups could help by distinguishing between lumbar stenosis (older population) and lumbar disc herniation (younger population), but the choice for younger and older than 55-years was made arbitrary. Moreover, we were not able to include variation in timing of surgical treatment for lumbar DDD in our analyses, since data on first visit to the General Practitioner (GP) were not available. Hereby, it is not possible to make strong conclusions on guideline implementation nor on the effect on quality of care. Also, disease severity might still explain a part of the observed variation for which we cannot account in this study. Our outcomes clearly show that variation between regions for these procedures was highly influenced by outliers such as postal code area ‘45’, in which patients probably receive surgical treatment in Belgium. Due to the (Dutch) boundaries of our administrative database, these procedures were not included in our database.

### Findings in relation to other studies

In 2013, a report was written on practice variation in the Netherlands between 2009 and 2011^23^. Ten-fold variation between regions was observed in surgical rates for patients suffering from lumbar disc herniations, with an interquartile range of 1.5. Removing outliers, we observed less than two-fold variation between regions and hospital service areas and a considerably lower interquartile range compared to the 2009–2011 data from the 2013 report.

### Clinical implications

The finding of low variation in laminectomies and discectomies for lumbar DDD suggests that the outcomes of the Sciatica trial were implemented nationwide. The delineated process concerning lumbar disc herniations serves as an exemplar of a knowledge and quality-of-care cycle. In this iterative cycle, high-quality clinical research initiates the formulation of guidelines. Subsequently, new knowledge gaps emerge during the guideline development process prompting revisions to the guidelines, and ultimately leading to their implementation. However, this study did not investigate the extent of dissemination of the guidelines, since we did not investigate potential factors influencing variation and no methodological methods or statistical analysis was used to evaluate (long-term) impact of guideline implementation.

Consequently, within the scope of the current study, it remains unclear regarding the extent to which the guidelines, alongside other factors, have contributed to the observed decrease in practice variation. Therefore, no causal effect of evidence-based research and guideline implementation on practice variation can be derived from the current results. Nevertheless, the finding of high variation for instrumented fusion surgery can be seen as such a knowledge gap and suggests that high-quality research on effectiveness is needed on this topic to improve quality of care for this patient group as well. In 2019, this was already stated at the Knowledge Agenda of the Dutch Association of Neurosurgeons (NvVN).

We observed that patient’s referral status was the most important factor for receiving surgical treatment. In the Netherlands, healthcare insurance companies head for regional collaboration between hospitals. The neurosurgical spine clusters are a good example of such a collaboration and the fact that referral status was such an important factor in our analyses matches this. The observation that opioid prescription emerged as a significant predictor for undergoing surgery may suggest increased severity of pain within this cohort. Nevertheless, given the recommended caution in prescribing opioids for chronic pain conditions, like lumbar stenosis, the correlation implies potential variability in opioid prescription practices, suggesting deviations from established guidelines. It is important to note, however, that the precise timing of opioid prescription remains unknown. This ambiguity could arise from the possibility of opioids being prescribed post-surgery, consequently indicating suboptimal outcomes and hampering the interpretation of this outcome. In literature, conflicting data on a higher risk for females on developing spondylolisthesis compared to males are described^24,25^. However, it was previously found that women have a slower rate of recovery and a higher risk of unsatisfactory outcomes after surgery, which might eventually lead to the need for fixation surgery^19^. The question arises as to why women exhibit an increased propensity for suboptimal outcomes following surgery. While empirical data to substantiate this notion are currently unavailable, one could posit that surgeons may defer surgical intervention in females, potentially resulting in a heightened susceptibility to unsatisfactory outcomes, as delineated in the study conducted by Bailey et al.^6^. This matches with the fact that we found females to have a lower risk of receiving a discectomy/laminectomy, but a higher risk of receiving instrumented fusion surgery compared to males.

## Conclusion

Our data revealed low regional and hospital variation in laminectomy and discectomy rates, but very high variation in instrumented fusion rates. Further studies are needed to investigate the extent of guideline implementation and its impact on practice variation.

### Supplementary Information

Supplementary Tables.

## Data Availability

The data that support the findings of this study are available from the Dutch Healthcare Authority Notably, the dataset used is not available for external parties. However, the data originate from non-public microdata obtained from Statistics Netherlands (CBS). Under certain conditions, these microdata are accessible for statistical and scientific research. For further information: microdata@cbs.nl.
