# Supplementary material for: Practice variation in surgical treatment for lumbar degenerative disc disease: exploring regional and hospital factors influencing surgical rates
Source: Sci Rep. 2024 Apr 23;14:9273. doi: 10.1038/s41598-024-59629-9 (PMC11039650; doi:10.1038/s41598-024-59629-9)
Supplement: Supplementary file 1 — Supplementary Tables. [file 41598_2024_59629_MOESM1_ESM.docx]

**Appendix tables**

Table 1. Appendix

| **Diagnosis treatment codes** | **Description** |
| --- | --- |
| ***Included*** |  |
| 0330-12-00-1203 | Pseudo radicular syndrome |
| 0330-12-00-1204 | Radicular syndrome |
| 0330-12-00-1211  0305-13-00-1350 | Spinal stenosis |
| 0305-13-00-1360 | Hernia Nuclei Pulposis |
| 0305-13-00-1370 | Spondylolisthesis |
| 0308-02-25-2501  0308-02-25-2550  0308-02-25-2565  0308-02-25-2555  0308-02-25-2505  0308-02-25-2510  0308-02-25-2515 | Degenerative disc disease |
| 0330-12-00-1231  0305-13-00-1330  0305-13-00-1340 | Low backpain |
| ***Excluded*** |  |
| 0330-12-00-1201  0330-12-00-1202  0330-05-00-0543  0305-12-00-1202  0305-12-00-1220  0305-12-00-1240  0308-02-25-2525  0308-02-25-2527  0308-02-25-2530  0308-02-25-2535 | Cervical diagnosis codes |
| 0330-02-00-0232  0330-02-00-0233  0330-02-00-0221  0330-02-00-0222  0330-02-00-0223  0308-02-21-2105  0308-02-21-2110  0308-02-21-2115 | Tumors, malignancies of the spine |
| 0308-02-24-2405  0308-02-24-2411  0305-13-00-1301  0305-13-00-1302 | Infectious spine disease |
| 0305-13-00-1396  0308-02-23-2305  0308-02-23-2311 | Spine trauma, fractures |
| 0305-13-00-1397  0305-13-00-1380  0305-13-00-1381  0305-13-00-1383  0305-13-00-1391 | Congenital disc disease, kyphosis, scoliosis |

Table 2. Appendix

| **Care product code** | **Description** |
| --- | --- |
| ***Included*** |  |
| 38437  38444  38438  38467 | Discectomy |
| 30327  30329 | Laminectomy |
| 38458  38459  38460  38464  38468  38469 | Fusion |
